# Large-scale comparative analysis of the nuclear factor-Y transcription factors across 320 horticultural and other plants

**DOI:** 10.1093/hr/uhaf304

**Published:** 2025-11-04

**Authors:** Kai Luo, Mingchao Li, Man Liu, Xitao Jia, Zhou Li, Xuechun Zhao, Jihui Chen, Xinyao Gu, Jin He, Chao Chen, Rui Dong

**Affiliations:** School of Tropical Agriculture and Forestry, Hainan University, Haikou 570228, China; School of Tropical Agriculture and Forestry, Hainan University, Haikou 570228, China; School of Tropical Agriculture and Forestry, Hainan University, Haikou 570228, China; Life Science and Technology School, Lingnan Normal University, Zhanjiang 524048, China; College of Animal Science, Guizhou University, Guiyang 550025, China; College of Animal Science, Guizhou University, Guiyang 550025, China; College of Animal Science, Guizhou University, Guiyang 550025, China; College of Animal Science, Guizhou University, Guiyang 550025, China; College of Agriculture, Key Laboratory of Plant Resource Conservation and Germplasm Innovation in Mountainous Region, Ministry of Education, Guizhou University, Guiyang 550025, China; College of Animal Science, Guizhou University, Guiyang 550025, China; College of Animal Science, Guizhou University, Guiyang 550025, China

## Abstract

Nuclear factor Y (*NF-Y*), evolutionarily conserved heterotrimeric transcription factors (TFs), are found throughout eukaryotic organisms. Comprising the *NF-YA*, *NF-YB*, and *NF-YC* subfamilies, this family is established as playing critical roles in plant growth and development. While earlier research has mainly centered on the functional and evolutionary characteristics of *NF-Y* within individual plant species, large-scale analyses and evolutionary patterns of these genes across major plant lineages remain largely unexplored. Here, we systematically identified 15 392 nonredundant genes of *NF-Y* family from 320 horticultural and representative plant species. Our findings showed that this gene family originated from charophytes. In bryophytes, pteridophytes, and gymnosperms, dispersed duplication served as the predominant mode of *NF-Y* gene expansion, whereas in angiosperms, their expansion was driven by whole genome duplication/segmental, dispersed, and tandem duplication. Conserved motif analysis revealed that highly conserved motifs are present within each *NF-Y* subfamily across eight representative plant species. However, some *NF-Y* genes in higher plants exhibited motif loss, indicating sequence variations during their evolutionary history. Transcriptomic profiling analysis of *NF-Y* genes in *Arabidopsis thaliana* under various conditions, including hormonal treatments, abiotic/biotic stresses, as well as various developmental stages, revealed their functional versatility. Furthermore, an interaction network comprising 36 *NF-Y* genes along with 2473 downstream and 261 upstream genes was constructed in *A. thaliana*. Enrichment analysis revealed interactions between *NF-Y* genes and other TFs, particularly those from the *Myb*_DNA-binding and APETALA2 (*AP2*) families, which were consistently enriched among both upstream and downstream regulatory genes. This work provides the first comprehensive and large-scale investigation into the evolutionary dynamics of *NF-Y* genes, encompassing taxa from basal algae to advanced horticultural plants, thereby offering novel insights into their evolutionary and lineage-specific expansion.

## Introduction

Plant development in natural environments is governed by diverse gene regulatory network and precisely regulated by numerous transcription factors (TFs) [1]. These TFs primarily regulate gene expression by binding promoter *cis*-acting elements or activating specific sequences that modulate transcriptional activity [2]. In plants, the complexity of TF regulatory networks arises from two key sources: the sheer abundance of TFs (reaching up to 2000 per genome) and their extensive diversity, spanning >50 families defined by DNA-binding domains [3]. Due to their central role in gene regulation, TFs offer promising targets for genetic manipulation to enhance crop productivity and improve resilience against biotic and abiotic stresses. Nuclear factor Y (NF-Y), alternatively termed CCAAT-binding factor (CBF) or heme activator protein (HAP), represents a ubiquitous and highly conserved family of TFs in eukaryotes. A defining feature of NF-Y is its selective binding to the conserved CCAAT motif in gene promoters [4]. The canonical NF-Y complex comprises three evolutionarily conserved subunits, namely *NF-YA* (also known as HAP2/CBF-B), *NF-YB* (HAP3/CBF-A), and *NF-YC* (HAP5/CBF-C), which assemble into a heterotrimer to perform their regulatory functions [5,6]. Among them, the *NF-YA* subunit contains critical domains for interactions with the other two subunits, as well as a DNA-binding domain that enables sequence specifically recognize of the CCAAT motif. Meanwhile, possessing a highly conserved histone-fold motif (HFM), *NF-YB* and *NF-YC* share structural features with histones H2B and H2A, respectively [7,8]. Within the *NF-Y* heterotrimer, *NF-YA* serves as the DNA-binding component, while the other two subunits stabilize the complex facilitate chromatin remodeling and recruit additional regulatory proteins [9,10]. In contrast to yeast and mammals, where each *NF-Y* subunit is typically encoded by a single gene, plant genomes possess multiple genes encoding members of each subunit category [11]. *Arabidopsis thaliana* harbors 36 *NF-Y* subunits (10 *NF-YAs*, 13 *NF-YBs*, 13 *NF-YCs*), while rice (*Oryza sativa*) contains 34 with 11 *NF-YAs*, 11 *NF-YBs*, and 12 *NF-YCs* subunits [12,13]. Given that the functional NF-Y complex typically comprises single copy from each subunit group, it is theoretically possible for a vast array of assemblies to theoretically. Recent phylogenomic evidence suggests that the evolutionary origin of NF-Y TFs traces back to early streptophyte algae, marking their emergence prior to the colonization of land [11]. During the transition to terrestrial environments, bryophytes, such as *Physcomitrium patens* expanded the NF-Y repertoire through dispersed duplication, resulting in multiple paralogs exhibiting divergent expression in response to desiccation and abscisic acid (ABA) signaling [14]. Similarly, in lycophytes and ferns, NF-Y members display structural conservation but increased regulatory diversity, reflecting early subfunctionalization of the three NF-Y subunits [8,15].

Recent research has highlighted the critical role of *NF-Y* TFs in plant organ development, as well as responses to biotic/abiotic stresses [6,16]. In *A. thaliana*, *AtNF-YB9* (*LEC1*) functions as a pivotal regulator in embryonic development and seed development, while *AtNF-YC3*, *AtNF-YC4*, and *AtNF-YC9* contribute to photomorphogenesis and seed dormancy [17–23]. In wheat (*Triticum aestivum*), *TaNF-YB1* interacts with *TaMADS29* to regulate kernel development [24]. *CmNF-YB8* gene in chrysanthemum (*Chrysanthemum morifolium*) could regulate flowering time by directly regulating cmo-miR156 [25]. Furthermore, *OsNF-YB1* has been shown to be essential for grain filling in rice by modulating the expression of sucrose transporter genes, whereas *OsNF-YA7* contributes to improved drought resilience via its involvement in the ABA-mediated signaling pathway [26,27]. *NF-Y* genes also contribute to symbiotic root nodule formation in legumes, where they participate in hierarchical transcriptional activation cascades within nodule morphogenetic signaling [28–31]. Moreover, *NF-Y* modulate plant-pathogen interactions, demonstrating their functional significance in immune mechanisms [32–34]. The results of these studies suggest that *NF-Y* members in plants might have functionally diverged.

During recent decades, a growing number of *NF-Y* homologous genes have been revealed in several single species, such as *Zea mays* [35,36], *Glycine max* [37], *Solanum tuberosum* [38], *Vitis vinifera* [39], *Brassica napus* [40], *Cucumis melo* [41], *Solanum lycopersicum* [42], *Prunus persica* [43], *Medicago sativa* [44], *Malus domestica* [45], *Populus trichocarpa* [46], and *Ginkgo biloba* [47]. While studies in a limited number of species suggest that *NF-Y* proteins originated before the emergence of land plants, no research to date has provided a comprehensive view of the genome-wide expansion and evolution of this gene family in other major plant lineages, such as bryophytes, pteridophytes, and algae. As more plant genomes become available, our understanding of the origin and evolution of *NF-Y* genes will continue to improve.

Thus, to improve knowledge on the evolutionary of the *NF-Y* family in plants, we conduct a large-scale comparative study of *NF-Y* gene in 320 species across the major plant lineages by using high-quality genomic data. Our analysis explores their evolutionary origins, expansion mechanisms, expression patterns, and regulatory networks, providing new insights into their functional roles. These findings offer a theoretical framework for future research and potential applications in crop improvement strategies.

## Results

### Identification and distribution of NF-Y family members across 320 species

**Figure 1:**
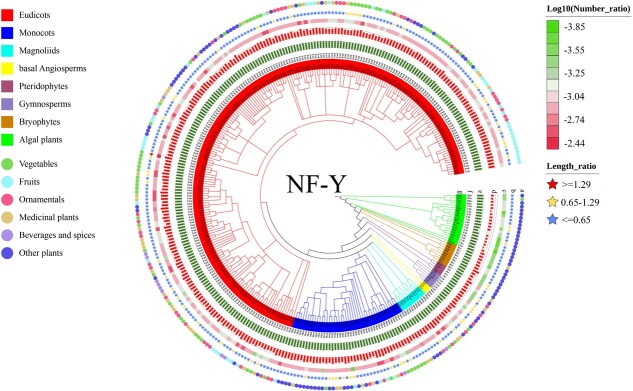
A comparative study on *NF-Y* gene families spanning 320 plant species. (a) Classification of 320 species, encompassing horticultural plants (fruits, vegetables, ornamentals, medicinal plants, and beverages and spices) alongside other representative species. (b) The ratio of the NF-Y protein length to the mean length of all proteins encoded in the genome of each species. (c) Log_10_ ratio of the number of *NF-Y* genes in each species to the number of protein-coding genes in its whole-genome. (d) Log_2_ value of the number of *NF-Y* genes in each species. (e) Log_10_ of the number of total protein-coding genes for each species. (f) Classification of each species. (g) The abbreviated Latin names of each species, with full names provided in Table S1.

To trace the evolutionary trajectory of plant *NF-Y* proteins, we analyzed 320 species representing major plant lineages. All selected species completed whole-genome sequencing and represent major plant taxa. The examined species comprised 20 algae and 300 land plants. The latter group included 223 eudicots, 44 monocots, 3 basal angiosperms, 11 magnoliids, 9 bryophytes, 6 gymnosperms, and 4 pteridophytes. Notably, more than 70% of these species were horticultural plants, including 47 fruit trees, 69 vegetables, 51 ornamental plants, 48 medicinal plants, and 11 beverage and spice plants (Fig. 1; Figs S1–S3; Table S1).

**Figure 2:**
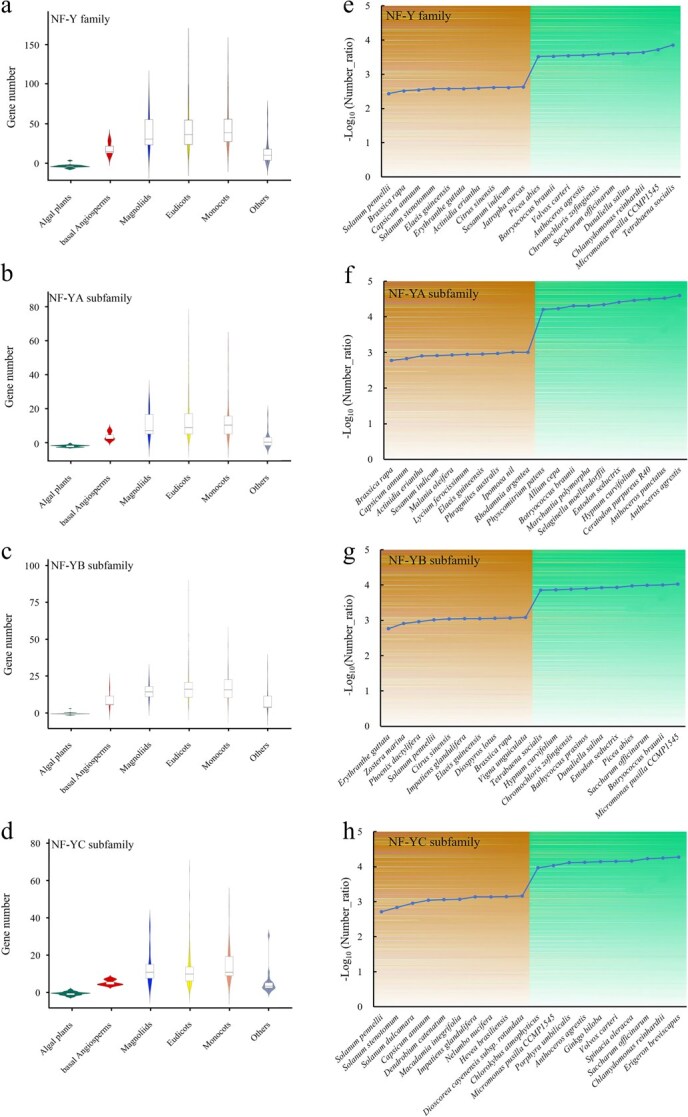
Comparative investigation of *NF-Y* gene families across plant species. (a–d) Distribution of *NF-Y* gene numbers among different categories of plants, visualized as a boxplot. (e–h) Comparison of *NF-Y* gene to protein-coding gene numbers in representative plants, expressed as a -log_10_ ratio. The ten species on the left of the horizontal axis correspond to those with the maximal ratio of identified NF-Y genes, whereas the ten species on the right correspond to those with the minimal ratio.

A total of 15 392 nonredundant *NF-Y* genes (4702 *NF-YA*s, 6302 *NF-YB*s, and 4388 *NF-YC*s) were identified across these 320 species based on the detection of conserved *NF-Y* domains (Fig. 1; Figs S1–S3; Tables S2–S6). A notable expansion in the number of genes encoding *NF-Y* orthologs was observed in land plants relative to algal species (Fig. 2a–d). The observed variation in gene copy number among land plants, ranging from 9 to 163, likely stems from episodic whole-genome or segmental duplications, coupled with subsequent gene loss events. The algal plants possessed 1 to 12 *NF-Y* genes, while only one such genes could be found in the *Picochlorum* sp. SENEW3, a unicellular aquatic alga. Despite this variability, all 320 examined species contained *NF-Y* gene, suggesting that the plant *NF-Y* family originated during the charophyte period.

Our study further compared per-species gene number for this family with total number of protein-coding genes in its genome (Fig. 2e–h; Tables S3–S6). Comparative analysis demonstrated expanded *NF-Y* gene numbers in land plants relative to algal lineages. The top 10 species by *NF-Y* gene abundance are all higher plants, containing nine eudicots (*Solanum pennellii*, *Brassica rapa*, *Capsicum annuum*, *Solanum stenotomum*, *Erythranthe guttata*, *Actinidia eriantha*, *Citrus sinensis*, *Sesamum indicum*, and *Jatropha curcas*) and one monocot (*Elaeis guineensis*) (Fig. 2e; Table S3). Conversely, 7 out of the 10 species with the lowest proportion of this family were classified as algal plants (Fig. 2e; Table S2). In *Tetrabaena socialis*, which had the lowest percentage of *NF-Y* family genes, only 2 *NF-Y* genes were identified among its 14, 296 total proteins, accounting for just 0.014% of its genome (Table S3). Similarly, the 10 species with the highest and lowest proportions of the 3 *NF-Y* subfamily members were shown in Fig. 2f–h, and Tables S4–S6. Notably, no *NF-YA* genes were detected in one rhodophyte (*Cyanidiococcus yangmingshanensis*) and seven chlorophytes (*Chlamydomonas reinhardtii*, *Dunaliella salina*, *T. socialis*, *Volvox carteri*, *Chromochloris zofingiensis*, *Micromonas pusilla* CCMP1545, and *Picochlorum* sp. SENEW3) (Table S4). Additionally, two chlorophytes (*T. socialis* and *Picochlorum* sp. SENEW3) lacked *NF-YC* genes (Table S6). Despite interspecific variation in the number of *NF-Y* gene copies, the number of *NF-Y* homologs in plant genomes is positively correlated with the total number of protein-coding genes according to Spearman correlation analysis (*r* = 0.655, *P* < 0.001) (Fig. S4).

Predicted *NF-Y* protein sequences across species typically ranged from 200 to 300 amino acids, beginning with an initiation codon and ending with a stop codon. The average length of *NF-Y* members exceeded those of all protein database sequences in only two species (*S. pennellii* and *Solanum pimpinellifolium*) (Table S3). Among the 3 *NF-Y* subfamilies, *NF-YA* genes were the longest on average (297.5 a.a.), followed by *NF-YC* (245.8 a.a.), while *NF-YB* genes were the shortest (190.3 a.a.) (Fig. 1; Table S3). Additionally, we analyzed and contrasted the average protein length of *NF-Y* members with the average lengths of all proteins within each species’ genome (Figs S1–S3; Tables S3–S5).

**Figure 3:**
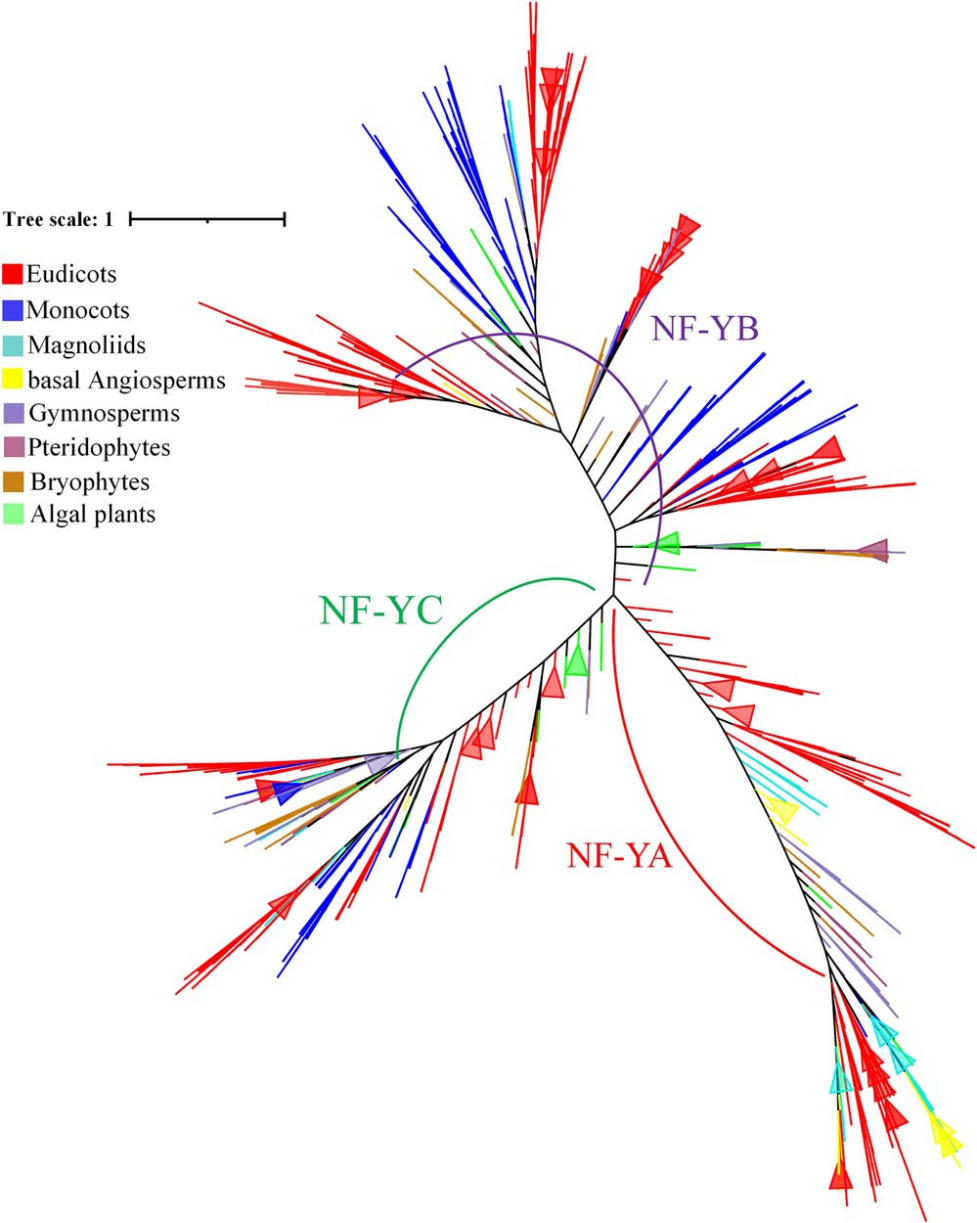
Phylogenetic analysis of the *NF-Y* gene family across 320 species. Amino acid sequences were aligned to construct phylogenetic tree using FastTree (v2.1.11).

To investigate the classification and evolutionary dynamics of *NF-Y* genes, we performed phylogenetic analysis based on 15 392 *NF-Y* sequences identified from 320 plant species (Fig. 3). The resulting phylogeny grouped *NF-Y* members into 3 main categories, *NF-YA*, *NF-YB*, and *NF-YC*, which match the known subfamily classifications. In each subfamily, most branches include *NF-Y* genes from diverse plant taxa, with those from algal plants located at the root of the phylogenetic tree. Interestingly, certain branches within the *NF-YA* and *NF-YB* subfamilies exclusively comprised monocot and dicot genes, with these lineages exhibiting clear phylogenetic separation. This pattern suggested that *NF-Y* genes evolved independently within each lineage following species divergence.

### Characterization of duplication types within the NF-Y family

**Figure 4:**
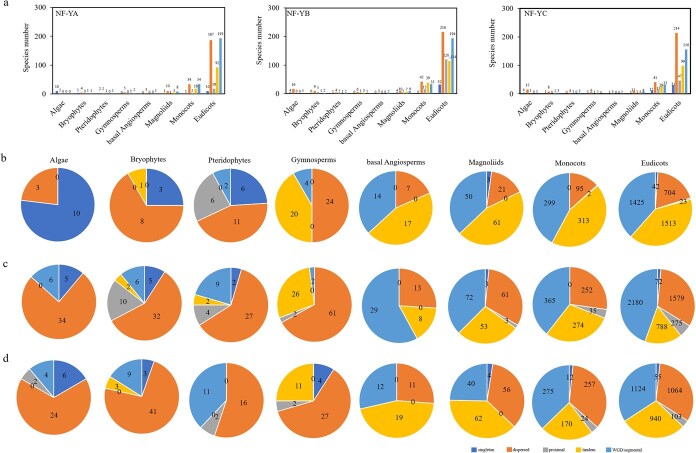
Duplication type of *NF-Y* family genes for 320 species. (a) The statistics of species with different duplicate models in *NF-Y* genes across different taxa. The number of species of each duplication type in *NF-Y* family in different evolutionary lineages was quantified. (b) The proportion of *NF-YA* subfamily genes relative to total duplication events was evaluated across distinct taxonomic groups. (c) The proportion of *NF-YB* subfamily genes relative to total duplication events was evaluated across distinct taxonomic groups. (d) The proportion of *NF-YC* subfamily genes relative to total duplication events was evaluated across distinct taxonomic groups.

In plant lineages, frequent gene expansion or duplication events are typically followed by sequence or functional divergence, contributing to subfunctionalization, the emergence of novel material, and potential adaptive benefits [48,49]. Multiple mechanisms have been proposed to account for gene duplication in plants, including whole genome duplication (WGD), segmental duplication, local events such as tandem and proximal duplications, as well as dispersed duplication [48–51]. The various duplication models differ in their gene retention tendencies, with some favoring the preservation of redundant copies, while others facilitate the emergence of novel functions and evolutionary diversification. To investigate *NF-Y* gene duplication across species, we inferred the underlying duplication mechanisms for all identified *NF-Y* family members in the analyzed genomes through MCScanX [52]. We further examined the distribution of species and genes within each *NF-Y* subfamily across these duplication modes. Overall, the expansion mechanisms of *NF-Y* genes varied across plant taxa (Fig. 4; Tables S7–S9). Within the *NF-YA* subfamily, algal species contained a higher proportion of singleton genes. In contrast, *NF-YA* genes in higher plants exhibited lineage-specific expansion patterns. Bryophytes and pteridophytes primarily expanded through dispersed duplication, while gymnosperms expanded through both dispersed and tandem duplication, and basal angiosperms expanded through both WGD/segmental and dispersed duplication. Within magnoliids, monocots, and dicots, *NF-YA* genes increased in number mainly by WGD/segmental duplication, and tandem duplication (Fig. 4a and b; Tables S7–S9). Dispersed duplication was identified as the primary driver of *NF-YB* subfamily expansion in algae, bryophytes, pteridophytes, and gymnosperms. In basal angiosperms, WGD/segmental and dispersed duplications played a major role, whereas magnoliids and monocots expanded through a combination of dispersed, WGD/segmental, and tandem duplication. In dicots, dispersed and WGD/segmental duplications were the predominant mechanisms (Fig. 4a and c; Tables S7–S9). A parallel pattern occurred in the *NF-YC* subfamily, with dispersed duplication serving as the predominant expansion mechanism for algal, bryophyte, pteridophytes, and gymnosperm lineages. Basal angiosperms primarily expanded through WGD/segmental and dispersed duplication. In evolutionary advanced angiosperm lineages (including magnoliids, monocots, and dicots), the expansion of *NF-YC* genes predominantly resulted from a combination of dispersed duplications, WGD/segmental duplications, and tandem duplication events (Fig. 4a and d; Tables S7–S9). Across all *NF-Y* subfamilies, relatively few genes were classified under the proximal duplication type (Fig. 4; Tables S7–S9), suggesting that this mechanism played a minor role in *NF-Y* family expansion.

### Loss and gain events of NF-Y genes during evolution

**Figure 5:**
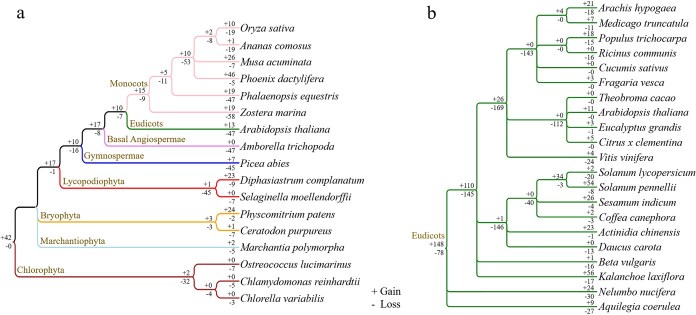
Loss and gain events of *NF-Y* genes in representative species using Notung software. (a) Analysis of gain/loss events within the *NF-Y* gene across representative species from diverse taxonomic groups. (b) Analysis of gain/loss within the *NF-Y* gene across representative eudicots. Branch-specific gain/loss events are denoted by numeric annotations (‘–’ = loss, ‘+’ = gain).

To further clarify the evolutionary trajectories of *NF-Y* genes, this study investigated events of duplication/loss across 17 representative species encompassing major evolutionary clades. These included three Chlorophyta, one Marchantiophyta, two Bryophyta, two Lycopodiophyta, one Gymnospermae, one basal Angiospermae, one eudicot, and six monocots (Fig. 5a).

Phylogenetic reconstruction revealed multiple independent and significant *NF-Y* gene loss/gain events throughout plant evolution. Within the ancestral lineage shared by these 17 plant species, a total of 42 *NF-Y* genes experienced duplication events, while no instances of gene loss were observed (Fig. 5a). However, subsequent evolutionary events varied across lineages. We found that an ancestor shared by three Chlorophyta species lost 32 genes but gained only two genes. Similarly, in the common ancestor of the two Bryophyta species, three genes were lost, while three were duplicated. In contrast, the lineage leading to bryophytes and other land plants underwent a total of 17 genes gain but only 1 gene lost (Fig. 5a). Despite widespread WGD and WGT events in land plants, *NF-Y* gene losses generally outnumbered gains in most representative species, except for *Diphasiastrum complanatum, Phoenix dactylifera, Musa acuminata,* and *P. patens* (Fig. 5a).

Moreover, we investigated the evolutionary trajectories of *NF-Y* members by assessing gene gain/loss events in a set of 21 representative eudicot genomes. The results show that the frequency of gene losses and gains varied among eudicot species (Fig. 5b). In summary, *NF-Y* gene gain and loss patterns did not show a clear correlation with life history traits or evolutionary distances among species. These findings suggest that the dramatic variation in *NF-Y* gene family size is driven by lineage-specific evolutionary processes.

### Conserved motif identification and distribution

**Figure 6:**
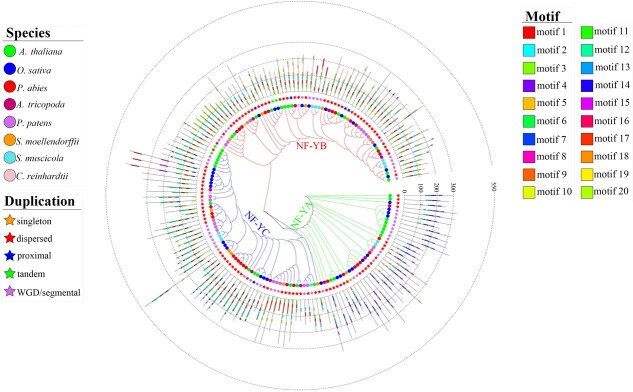
Analyses of evolutionary patterns, including phylogeny, motif conservation, and gene duplication events, were performed for *NF-Y* family genes across eight representative plant species. Phylogenetic reconstruction of *NF-Y* genes was performed using FastTree with a maximum-likelihood algorithm based on their protein sequences. Conserved motifs were characterized via the MEME, and gene duplication types were inferred using MCScanX.

To deepen our understanding of phylogenetic relationships among *NF-Y* gene, we performed an analysis of conserved motifs. Eight representative plant species from different evolutionary lineages, ranging from algal plants to land plants, were selected. These included two chlorophyte algae (*Spirogloea muscicola* and *C. reinhardtii*), one moss and one lycophyte (*P. patens* and *Selaginella moellendorffii*), one gymnosperm (*Picea abies*), one basal angiosperm (*Amborella trichopoda*), and two angiosperms, comprising the model eudicot *A. thaliana* and the model monocot *O. sativa*. In total, 161 *NF-Y* family genes were identified across these eight species. Among the surveyed species, *A. thaliana* harbored the highest number of *NF-Y* genes (36), followed by *O. sativa* (34) and *P. patens* (22) (Fig. 6). All eight representative plant species contained entire three *NF-Y* subfamilies, except for *C. reinhardtii*, of which no *NF-YA* subfamily members were detected in this species.

A total of 20 specific motifs (motifs 1–20) were estimated using MEME software (Fig. 6; Figs S5 and S6; Table S10). The types and positions of conserved motifs were largely consistent across different *NF-Y* subfamilies, aligning with their phylogenetic relationships. Among the three subfamilies, *NF-YA* proteins were the most conserved, containing nine motifs. In contrast, *NF-YC* proteins were the most diverse, containing up to 13 motifs. Almost all *NF-YA* subfamily members harbored motifs 2, 4, and 8, with motifs 4 and 8 being exclusively found in this group as distinguishing features. These motifs were annotated as components of the CBF-B/NF-YA subunit (a CCAAT-binding TF), indicating its potential role in the CBF domain. Motifs 1 and 3 occurred solely in both *NF-YB* and *NF-YC* subfamilies. Motif 1, annotated as a histone-like TF of the *CBF/NFY* family and archaeal histone, was consistently identified across all *NF-YB* and *NF-YC* proteins, suggesting conserved functional characteristics and potential evolutionary convergence between these two subfamilies (Fig. 6; Figs S5 and S6; Table S10).

We also investigated the evolutionary history of conserved motifs in *NF-Y* family proteins (Fig. 6; Fig. S6; Table S10). Eight conserved motifs (motifs 1–6, 8, and 13) were detected in at least one member of the *NF-YA*, *NF-YB*, or *NF-YC* subfamilies across all eight representative species, although certain motifs were absent in specific evolutionary lineages. Such as, in *NF-YB* subfamily, motif 3 was fully conserved in *A. thaliana, O. sativa*, *P. patens*, and *S. moellendorffii*, but was partially lost in *P. abies*, *A. trichopoda*, *S. muscicola*, and *C. reinhardtii*. Motif 13 was consistently identified in the *NF-YA* subfamily across all eight species, although it was absent in certain protein sequences, such as *AthNF-YA2* and *AthNF-YA10* in *A. thaliana*. Additionally, the remaining 12 motifs were presented in some proteins of certain species, but was completely lost in other species. Notably, two genes (*OsaNF-YA8* and *AtrNF-YC5*) had the highest number of missing motifs, each retaining only one motif, suggesting a potential loss of function during evolution (Fig. 6; Fig. S6; Table S10).

Comparative motif analysis revealed progressive changes in *NF-Y* subunit composition across plant lineages (Fig. 6; Fig. S6; Table S10). Motifs 7, 9, 11, 15, and 17 exhibited an identical distribution pattern, being entirely absent from all examined charophyte species. Motifs 15, 16, and 17 were entirely missing in the *P. patens*, while *S. moellendorffii* and *P. abies* exhibited a relatively high level of motif depletion, losing eight and five motifs, respectively. In contrast, angiosperms displayed more conserved motif profiles, with only three to four motifs completely lost across these lineages. Remarkably, *O. sativa* uniquely lacked motif 10 and motif 11, a pattern distinct from other angiosperms, suggesting divergent evolutionary trajectories in monocot *NF-Y* gene regulation (Fig. 6; Fig. S6; Table S10). Overall, these results indicate that sequence divergence has occurred in genes of this family during evolutionary time, contributing to functional diversification across plant lineages.

### Gene expression profiling of the NF-Y family under multiple conditions.

**Figure 7:**
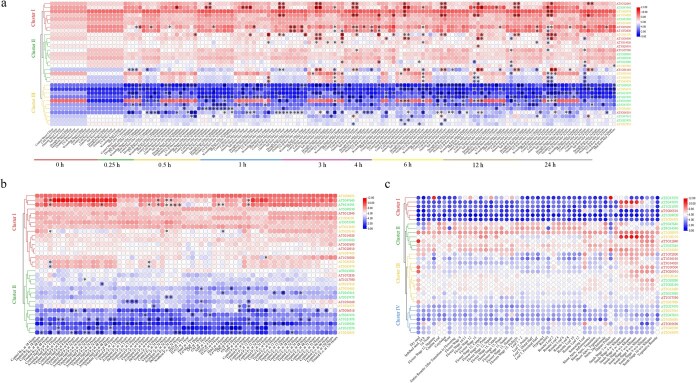
Expression profiles analysis of *NF-Y* genes in the *A. thaliana*. (a) Transcript data of *NF-Y* genes under diverse abiotic stresses was assessed through absolute quantification. (b) Transcript data of *NF-Y* genes under diverse biotic stresses was assessed through absolute quantification. (c) Transcript data of *NF-Y* genes were examined across multiple developmental stages and tissue types. All expression values in the heat map are log2-transformed. Samples subjected to abiotic or biotic stress were marked with an asterisk if their gene expression showed a fold change (FC) of ≤0.5 (downregulation) or ≥ 2 (upregulation) compared to the corresponding control groups.

To investigate the expression profiles of *NF-Y* genes across diverse conditions, we analyzed comprehensive expression data obtained from the *Arabidopsis* eFP database. A heatmap was generated to illustrate *NF-Y* gene expression across various treatments (Fig. 7; Fig. S7; Tables S11–S14). A total of 154 samples from 18 groups were analyzed to examine *NF-Y* gene expression under various abiotic stress conditions. Expression levels were assessed in both shoots and roots at multiple time points (0, 0.25, 0.5, 1, 3, 4, 6, 12, and 24 hours) (Fig. 7a; Table S11). Cluster analysis revealed three distinct expression patterns: genes in Cluster I exhibited consistently higher expression levels across most stress conditions, whereas those in Cluster III showed generally lower expression. However, one *NF-YB* subfamily gene, *AT4G14540*, deviated from the Cluster III pattern, maintained consistently higher expression in shoots compared to roots in many stress treatments. To further explore *NF-Y* gene responses to biotic stress, we analyzed expression data from 70 samples spanning 27 experimental conditions (Fig. 7b; Table S12). Cluster analysis separated the samples into two major groups based on gene expression patterns. Genes in Cluster I generally exhibited elevated expression in response to biotic stress (Fig. 7b; Table S12).

The transcript data expression patterns of *NF-Y* genes were analyzed during various growth phases and in multiple organs, such as leaves, roots, flowers, seeds, and siliques (Fig. 7c, Table S13). Cluster analysis revealed four major groups: genes in Cluster I exhibited lower expression across most tissues, while those in Cluster II had relatively higher expression. Interestingly, *AT2G13570*, an *NF-YB* subfamily gene in Cluster I, showed elevated expression specifically in stamens at flower stage 12 and in mature pollen. Another *NF-YB* subfamily gene in Cluster I, *AT5G47670*, displayed high expression levels in siliques during seed development stages 4–8, suggesting a potential role in reproductive development (Fig. 7c, Table S13). We analyzed expression data from 81 samples subjected to different hormone treatments, including ACC, zeatin, IAA, ABA, MJ, GA-3, and BL (Fig. S7, Table S13). Heatmap analysis revealed distinct expression patterns, with *NF-Y* genes clustering into four groups. Clusters II and III contained ten genes with relatively high expression, while Cluster I comprised eight genes with lower expression levels (Fig. S7, Table S14). Analysis of *A. thaliana* expression data revealed several prominently expressed core genes, such as *AT1G08970*, *AT3G53340*, *AT5G47640*, and *AT4G14540*, which may play key roles in stress responses and the regulation of growth and developmental processes. These expression profiles in *A. thaliana* provide a valuable reference for understanding *NF-Y* gene function in other plant species and can guide future functional studies.

### Identification of upstream and downstream genes and regulatory network construction

**Figure 8:**
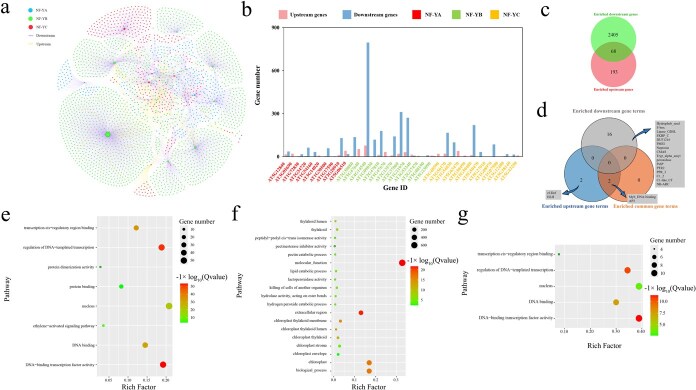
The interaction landscape of *NF-Y* genes in *A. thaliana*, encompassing their upstream regulatory elements and downstream transcriptional targets. (a) The interaction network was visualized and analyzed using Gephi software. (b) The count of upstream regulators and downstream targets associated with each *NF-Y* gene within the interaction network. (c) Specific and shared *NF-Y* genes between upstream regulators and downstream targets in the interaction network. (d) Pfam terms uniquely and enriched among upstream regulators, downstream targets, and commonly shared gene sets. (e–g) GO enrichment analysis of the upstream regulators, downstream targets, and the common genes, respectively. Pfam and GO annotation terms exhibiting significant enrichment, defined by *P* < 0.05 and false discovery rate (FDR) < 0.05 thresholds.

The regulatory mechanisms governing the *NF-Y* gene family, including its upstream regulators, downstream targets, and the extent of functional redundancy and specificity, remain largely unclear. To clarify the regulatory landscape of the *A. thaliana NF-Y* gene family, we retrieved upstream and downstream genes using the integrated Gene Regulatory Network (iGRN) database (Tables S15, S16) and constructed a regulatory network (Fig. 8a). This network included 3500 gene pairs, comprising 2473 downstream genes regulated by *NF-Y* genes and 261 upstream regulators of *NF-Y* genes (Fig. 8b; Tables S15, S16). Our analysis revealed substantial variation in the number of downstream targets associated with individual *NF-Y* regulators. Among the upstream regulators, *AT4G14540* from the *NF-YB* subfamily was associated with the largest number of upstream regulators (78), while *AT1G54160* from the *NF-YA* subfamily and *AT5G50470* from the *NF-YC* subfamily had the fewest (1). Notably, no upstream regulators were identified for *AT5G27910* and *AT1G07980*, both from the *NF-YC* subfamily (Tables S15 and S16). For the downstream genes, *NF-YB* member *AT4G14540* displayed the most extensive regulatory influence (796 target genes), contrasting with *NF-YA* component *AT3G05690* which affected only a single target. Interestingly, eight *NF-Y* genes had no identified downstream targets, including *AT5G47640*, *AT5G47670*, and *AT2G27470* from the *NF-YB* subfamily, as well as *AT3G48590*, *AT5G63470*, *AT5G50490*, *AT1G07980*, and *AT5G43250* from the *NF-YC* subfamily. In addition, 68 genes were found to function both as upstream regulators and downstream targets of *NF-Y* genes, suggesting a potential role in feedback control pathways (Fig. 8C; Tables S15 and S16).

For functional characterization of these genes, we performed Pfam domain, Gene Ontology (GO), and Kyoto Encyclopedia of Genes and Genomes (KEGG) pathway enrichment analyses (Fig. 8d–g; Fig. S8; Tables S17–S19). Pfam domain analysis revealed that upstream regulators were largely enriched in TF families including APETALA2 (*AP2*), *Myb*_DNA-binding, and helix–loop–helix. Significant overrepresentation was observed among downstream genes encoding Hydrophob_seed, F-box, and GDSL-like Lipase/Acylhydrolase family proteins, highlighting their crucial involvement in key developmental transitions, such as embryo development and seed germination (Fig. 8d; Table S17).

GO analysis revealed that upstream regulators were mainly implicated in processes, such as transcriptional regulation mediated by DNA templates, TF activity involving DNA binding, and ethylene-responsive signaling. In contrast, downstream targets exhibited significant enrichment in pathways related to lipid degradation, hydrogen peroxide catabolic process, and pectin metabolism, highlighting their functional roles in various metabolic processes. Genes functioning both upstream and downstream were significantly enriched in the DNA binding and nucleus category (Fig. 8e–g; Table S18), which is consistent with the TFs and their functional descriptions. KEGG pathway enrichment analysis further revealed that downstream *NF-Y* target genes were significantly enriched in pentose and glucuronate interconversions, and tropane, phenylpropanoid, piperidine, and pyridine alkaloid biosynthesis (Fig. S8; Table S19), indicating a potential regulatory role for *NF-Y* genes in diverse plant metabolic processes, whereas upstream genes exhibited no significant enrichment in any specific pathway. These findings offer significant understanding of *NF-Y* gene regulatory mechanisms in *A. thaliana*, revealing complex upstream and downstream regulatory relationships. The presence of feedback-regulated genes and their enrichment in key functional categories demonstrate the critical role of *NF-Y* genes in plant transcriptional regulation, developmental processes, and stress responses.

### Dual-luciferase assay confirmed the *AtNF-Y2* interaction of *AtERF115*

**Figure 9:**
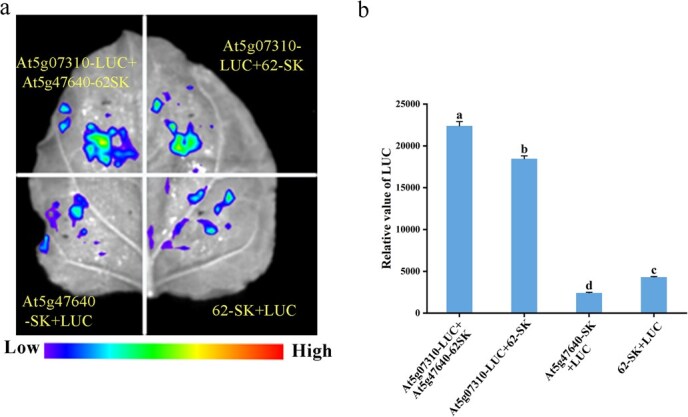
Interaction verification between *At5g07310* and *At5g47640*. (a) Typical imaging results for *N. benthamiana* leaves following 48-hour infiltration. (b) Relative LUC activity with respect to normalized REN luciferase activity. Values represent the means ± SE from six biological replicates. Statistically significant differences, as evaluated by Duncan’s multiple range test (*P* < 0.05), are represented by different letters.

Members within the ethylene response factor (*ERF*) TF family, part of the *AP2/ERF* superfamily, are well known for mediating plant growth, development, and defense through modulating ethylene biosynthesis and signaling [53]. A dual-luciferase reporter assay was performed to assess whether *At5G47640* (*NF-YB2*) is regulated by *At5G07310* (*ERF115*). Co-expression of *At5G07310*-LUC with *At5G47640*-62SK in *Nicotiana benthamiana* leaves resulted in significantly stronger LUC luminescence signals compared to the negative control (co-expression of *At5G07310*-LUC with the empty 62SK vector) (Fig. 9a). No significant differences were observed between the control groups (*LUC + 62SK* and *At5G47640*-SK + LUC). This result was further supported by relative LUC/REN activity measurements (Fig. 9b). Together, these findings demonstrate that *At5G07310* positively regulates *At5G47640* in vivo, suggesting direct regulatory interaction between *NF-Y* and *ERF* gene families.

## Discussion

Research on *NF-Y* genes in plants has expanded significantly since their discovery [22,54]. These genes have been discovered across a wide range of plant taxa, encompassing both monocotyledonous and dicotyledonous species [12,13,35]. Prior investigations into the phylogenetic relationships of the *NF-Y* gene have significantly advanced our understanding of its evolutionary development [16]. Due to the sparse inclusion of early-diverging lineages such as basal angiosperms, gymnosperms, and non-vascular plants, significant gaps remain in clarifying the evolutionary trajectory, diversification, and phylogenetic context of the *NF-Y* gene family across land plants and algae. With advancements in sequencing technology and decreasing costs, vast amounts of high-quality genome data have become available, which offers an excellent opportunity for large-scale plant TF families studies [55].

Previous research has indicated that while animals and fungi each contain a single *NF-Y* subunit, plants possess multiple subunits [11]. In this study, we analyzed 15 392 *NF-Y* sequences from 320 plant species, including 20 algae and 300 land plants. Most angiosperms possess a higher number of *NF-Y* genes compared to other plant lineages, likely due to historical genome duplication events that have contributed to gene family expansion. For example, *S. pennellii*, a wild tomato species, exhibited the highest proportion of *NF-Y* family genes, suggesting the *NF-Y* family may have crucial and special roles in this species. In contrast, *Picochlorum* sp. SENEW3 contained only a single *NF-Y* gene. It is well established that all three *NF-Y* subunits are essential for CCAAT box recognition; *NF-YA* establishes targeted binding, whereas *NF-YB* and *NF-YC* confer complex stability [4,56]. Thus, *NF-Y*-mediated stable DNA binding necessitates a complete subunit assembly [10]. At *NF-YA2* has been identified to directly recognize the NFYBE *cis*-element (not the *CCAAT* box), while specific *NF-YC* members have been shown to associate with HISTONE DEACETYLASE 15 (HDA15) [57,58]. We speculate that this gene loss may lead to functional divergence within this family, potentially resulting in alternative regulatory mechanisms through binding to alternative *cis*-regulatory elements. Further research will likely uncover novel mechanisms in these lineages. Significantly, charophytes were identified as the earliest *NF-Y*-containing algae in our study, supporting the hypothesis of this clade as the gene family’s likely birthplace. Based on phylogenetic reconstruction, 15 392 *NF-Y* genes from 320 species were classified into three distinct subfamilies, highlighting their strong intragroup evolutionary relatedness. From a structural perspective, *NF-YA* harbors two conserved *α*-helices, whereas *NF-YB* and *NF-YC* both exhibit histone-fold structures. However, their roles in DNA recognition diverge, with *NF-YB*’s α1 helix playing a pivotal role in binding DNA, and *NF-YC* contributing minimally [11,16]. Such fundamental structural differentiation likely underlies their distinct evolutionary trajectories, ultimately resulting in the clear phylogenetic separation of these three subfamilies.

The occurrence of genome duplications has been a key driver in plant evolutionary history, contributing significantly to both the diversification and retention of gene families. These events were critical for the growth and adaptability of plants under varying stress conditions [59,60]. Current investigations into plant gene duplication have delineated diverse duplication mechanisms [61]. Research demonstrated that *NF-Y* genes in *P. trichocarpa* and *S. tuberosum* originated from tandem and WGD/segmental events [38,46]. Evidence indicates taxon-dependent drivers underlying the focal family's expansion, with mechanisms differing from basal aquatic flora to derived terrestrial plants. In algae, bryophytes, and pteridophytes, *NF-Y* gene expansion occurred predominantly via dispersed duplication. Conversely, among basal angiosperms, magnolids, monocots, and eudicots, expansion was primarily attributable to WGD, dispersed duplication, and tandem duplication. WGD or polyploidy was a common feature of plant genomes, resulting in variations in genome size and content [62]. Expansion of TF lineages across seed plants was principally fueled by ancient WGD events, as revealed here [63]. Significantly, gene family expansion in numerous species predominantly relied on dispersed duplication, a mechanism equally pivotal for *NF-Y* family enlargement [51,64]. This underscores a unique evolutionary trajectory for this plant gene family's expansion.

For deeper evolutionary insights into *NF-Y* subfamily genes in representative plants, we carried out phylogenetic and conserved motif analyses. Phylogenetic analysis showed that each *NF-Y* subfamily member retained the conserved domains and motif compositions characteristic of its corresponding subunit, confirming the high conservation of *NF-Y* in eukaryotes [65]. Previous research suggested that some *NF-Y* family genes, such as *AtNF-YB11/12/13* and *AtNF-YC10/11/13*, might not belong to the *A. thaliana NF-Y* family due to lacking the proper structural features [66]. Our phylogenetic tree analysis supports this observation. Certain *NF-Y* members, especially from *A. trichopoda*, *P. patens*, and *P. abies*, exhibited a more distant evolutionary relationship with the *NF-YB* and *NF-YC* clusters, similar to the *A. thaliana NF-YB11/12/13* and *NF-YC10/11/13* genes. Motif analysis revealed that motif 8 in the *NF-YA* subfamily is associated with DNA targeting, while motifs 2, 3, and 4 in all subfamilies appear to be involved in interactions with other proteins, suggesting their role in protein–protein interactions within the *NF-Y* family.

Despite widespread recognition of *NF-Y* genes as central regulators of plant development and stress responses, the molecular mechanisms underlying their functions remain largely unresolved, and comprehensive genome-scale analyses of their binding landscapes in plants are still scarce. For deeper insights into *NF-Y* gene functions in plants, we analyzed public transcript profiling data (eFP Browser) in *A. thaliana* under various conditions. Our findings revealed significant variability in *NF-Y* gene expression profiles, indicating their diverse functional involvement during developmental processes and stress adaptation. Accumulating evidence indicates that elevated expression of specific *NF-Y* subunits, including *NF-YB2/3* and *NF-YC1/3/4/9*, plays a regulatory role in the control of flowering time [20,67,68]. In this study, transcript profiling data analysis revealed that numerous *NF-Y* genes exhibited high expression levels in floral tissues across multiple developmental stages. Notably, a greater proportion of genes exhibited elevated expression levels in response to stress, which may be indicative of functional divergence driven by evolutionary processes. Certain genes exhibited condition-specific expression. For instance, *AT5G47670* (*AtNF-YB6*) exhibited high expression in seed-stage siliques, suggesting involvement in embryo development, whereas *AT5G47640* (*NF-YB2*) displayed elevated expression in shoots under osmotic and salt stress, in agreement with prior reports [15,69,70]. The transcriptome-based evidence lays a solid groundwork for advancing functional characterization of *NF-Y* genes across diverse plant systems. A regulatory network of *NF-Y* gene interactions was established via the iGRN database, supplemented with comprehensive enrichment analyses [71]. This network, comprising 3500 gene pairs (2473 downstream and 261 upstream genes), revealed complex regulatory relationships. Upstream *NF-Y* genes were primarily associated with TF families including *AP2*, *Myb_DNA-binding*, *zf-Dof*, and *HLH*, implying functions related to multiple stress conditions. These findings align with earlier studies demonstrating *A. thaliana NF-YC*-mediated flowering via SOC1 induction under drought through ABA-responsive element (ABRE)-binding factor (ABF) interaction [72], and *PdNF-YB21*’s role in drought tolerance via interaction with PdFUSCA3 (B3 domain) in *Populus* [73]. Interestingly, genes functioning both upstream and downstream of *NF-Y*s were co-enriched in *AP2* domains, which are associated with ethylene signaling [55]. This suggests functional interaction between the *NF-Y* and *ERF* families. Although evidence for such interaction exists in rice [74], studies in *A. thaliana* remain limited. Our dual-luciferase reporter assay confirmed an in vivo interaction between *AtNF-YB2* and *AtERF115*, validating predictions from the iGRN database and supporting previous findings [71]. *AtERF115* is known for its role in wound-induced responses [75]. Thus, the interaction suggests that *AtNF-YB2* may contribute to tissue repair and organ regeneration. This function could play a significant role in horticultural plants by enhancing their regenerative capacity, which is crucial for vegetative propagation and stress recovery. In summary, our comprehensive analysis in *Arabidopsis* provides valuable insights into the evolutionary history, regulatory mechanisms, and functional diversity of *NF-Y* genes, offering a strong foundation for future studies on their roles in horticultural crops.

## Conclusion

This study systematically identified and analyzed *NF-Y* TFs across 320 plant species, including over 200 horticultural crops and several basal lineages. Comparative genomic and phylogenetic analyses revealed that *NF-Y* genes originated in streptophyte algae and gradually expanded in bryophytes and pteridophytes through dispersed duplication. In angiosperms, particularly eudicots and monocots, large-scale expansion mainly resulted from whole-genome and tandem duplications. Notably, significant *NF-YB* and *NF-YC* expansions occurred in *S. lycopersicum*, *V. vinifera*, and *C. melo*, likely reflecting adaptive evolution related to reproductive development, fruit ripening, and abiotic stress tolerance. Similar diversification in woody crops such as *M. domestica* and *P. persica* may support perennial growth and organ differentiation. Overall, this work establishes the broad evolutionary framework of *NF-Y* TFs across plant lineages, providing essential genomic resources for exploring their lineage-specific functional divergence. The results offer valuable guidance for future functional genomics and molecular breeding in horticultural species, especially in deciphering how *NF-Y* expansions contribute to developmental plasticity and environmental adaptability.

## Materials and methods

### Retrieval of sequences

We selected 320 plant species spanning eight major lineages, from algal plants to land plants (bryophytes, pteridophytes, gymnosperms, basal angiosperms, magnoliids, monocots, and eudicots). For each lineage, at least three to four species with relatively well assembled and annotated genomes are chosen as representatives. Amino acid sequences of protein-coding genes and their corresponding annotation files were retrieved from publicly available databases. A detailed species list provided in Table S1.

### Identification of NF-Y family members in multiple species

For screening potential *NF-Y* genes throughout the genome, we implemented a dual-approach strategy using BLAST and Hidden Markov Model (HMM) searches. Firstly, 36 *NF-Y* protein sequences derived from *A. thaliana* [12] served as queries for TBLASTP across all species genomes, utilizing TBtools-II.v2.119 [76]. Secondly, the HMM profile corresponding to the *NF-YA* (PF02045) and *NF-YB/C* (PF00808) was retrieved from Pfam (http://pfam-legacy.xfam.org) for querying predicted protein sets across all species, employing the HMMER package v3.2.1[77]. Proteins meeting an e-value cut-off of 1e^−4^ were retained as potential candidates. The final candidate set was determined by taking the intersection of hits from both methods, ensuring higher confidence in gene identification. Each candidate protein was examined for conserved *NF-Y* domains with online tools InterPro (https://www.ebi.ac.uk/interpro/entry/pfam/) [78] and NCBI Batch CD-search (https://www.ncbi.nlm.nih.gov/Structure/bwrpsb/bwrpsb.cgi) [79]. Classification of each candidate sequence into *NF-YB* and *NF-YC* subfamilies was performed based on their most significant BLAST hits. Candidates were assigned to a subfamily when at least two of their three most significant BLAST matches belonged to the same subfamily. Finally, we manually verified each classified sequence using DNAMAN software, following the plant-specific domain architecture criteria established by Petroni *et al*. [16].

### Analysis of duplication types in NF-Y genes

MCScanX was used to identify and analyze gene duplication types [52]. BLASTP (e-value 1 × 10^−5^) was used to compare protein sequences. Detection of collinear blocks was performed with default parameters. Using the duplicate_gene_classifier program, the gene duplication pattern was classified as singleton, dispersed, tandem, proximal, or segmental/WGD, based on copy number and genomic distribution.

### Phylogenetic reconstruction and motif analysis

Alignment of identified NF-Y protein sequences utilized MUSCLE v3.8.31 at default parameters [80]. FastTree v2.1.11 was used to construct the phylogenetic tree with default parameters [81]. iTOL v6 was employed for visualizing the constructed phylogenetic trees [82]. Gene gain and loss events at divergence nodes were inferred using Notung software [83]. Species trees were generated using NCBI Taxonomy Browser. To evaluate the structural integrity of the candidate NF-Y genes, conserved motifs were detected using the MEME suite (http://meme-suite.org/tools/meme), with: maximum 20 motifs, width 11–50 amino acids [84]. Redundant motifs were merged using Tomtom (*P* value <1e^−5^), and the final set was curated based on functional annotations.

### Analysis of gene expression under different conditions

The transcriptomic data for various stresses and developmental stages were sourced from the *A. thaliana* eFP Browser microarray datasets. This dataset comprises 18 groups of 154 samples under distinct abiotic stresses, 27 groups of 70 samples under distinct biotic stresses, 47 samples representing different developmental stages, and 10 groups of 81 samples under distinct hormone treatments [85]. These datasets served for *NF-Y* gene family expression analysis, with all relevant experimental details incorporated in the text boxes of the *Arabidopsis* eFP browser. Under the ‘absolute’ expression analysis framework, each gene’s expression across sample/condition was normalized by its own highest detected signal intensity, ensuring comparability. Clustered expression data were visualized as a heatmap using TBtools-II.v2.119 [76]. All expression values were log_2_ transformed. In the heatmaps, samples subjected to abiotic or biotic stress were marked with an asterisk if their gene expression showed a fold change (FC) of ≤0.5 (downregulation) or ≥2 (upregulation) compared to the corresponding control groups.

### Target gene identification, interaction network construction, and functional annotation

Target genes of the *NF-Y* gene family in *A. thaliana* were identified through the iGRN database, applying a stringent confidence score threshold of ≥0.60 [71]. In this study, target genes were divided into downstream and upstream genes. Gephi software (v0.9.2) constructed the interaction network between *NF-Y* family genes and their target genes, employing the ForceAtlas2 continuous graph layout algorithm (https://gephi.org) [86]. Functional enrichment analyses of SMART, GO, and KEGG were carried out in TBtools-ll.v2.119 [76], based on annotations generated by eggnog-mapper v2.0 [87]. Statistically significant enrichment terms were identified by applying thresholds of *P* < 0.05 and false discovery rate (FDR) < 0.05 [88].

### Reporter gene analysis using dual-luciferase

Given the significant enrichment of *AP2* domains among *NF-Y* upstream regulators, we hypothesize a potential interaction between *NF-Y* and *ERF* gene families. To test this hypothesis, we selected *AT5G47640* (annotated as *AtNF-YB2*), one of the highly expressed core *NF-Y* genes identified above, and its putative upstream regulator *AT5G07310* (annotated as *AtERF115*) based on the iGRN database. Confirmation of the interaction between *AtERF115* and *AtNF-YB2* was achieved via a dual-luciferase (LUC) transient expression assay. The complete coding sequence (CDS) of *AtNF-YB2* was inserted into the pGreenII 0800-LUC vector. This vector harbors a Renilla luciferase (REN) gene under the control of the CaMV 35S promoter. *ERF115* coding sequence (CDS) was cloned into the pGreenII 62-SK vector, creating the effector plasmid 62-SK-*ERF115*. The empty pGreenII 62-SK vector served as the negative control. For reporter gene assays, the promoter sequences of both genes were subcloned into the pGreenII 0800-LUC vector. All plasmid constructs were introduced into *Agrobacterium tumefaciens* GV3101 (pSoup), after which combinations of effector and reporter vectors were co-delivered into *N. benthamiana* leaves via agroinfiltration. After 48 hours, LUC activity was visualized (NightSHADE LB 985, Berthold), and dual-luciferase activity was quantified (Dual-Luciferase® Reporter Assay System, Promega, E1910) Six biological replicates were used for each experiment.

### Statistical analysis

Data were analyzed using SPSS 21.0 software (IBM Corporation, Armonk, NY, USA). Results are presented in the form of mean ± standard deviation. For multiple comparisons, the Duncan’s method was applied following ANOVA. To evaluate the relationship between *NF-Y* gene numbers and total protein-coding gene counts in plant genomes, a Spearman correlation analysis was performed. A threshold of *P* < 0.05 was considered indicative of statistical significance throughout the analysis.

## Supplementary Material

Web_Material_uhaf304

## Data Availability

All materials and related datasets in this study are available in Table S1.
