# Supplementary material for: Large-scale comparative analysis of the nuclear factor-Y transcription factors across 320 horticultural and other plants
Source: Hortic Res. 2025 Nov 4;13(2):uhaf304. doi: 10.1093/hr/uhaf304 (PMC12936444; doi:10.1093/hr/uhaf304)
Supplement: Web_Material_uhaf304 [file web_material_uhaf304.zip › Fig S1.pdf]

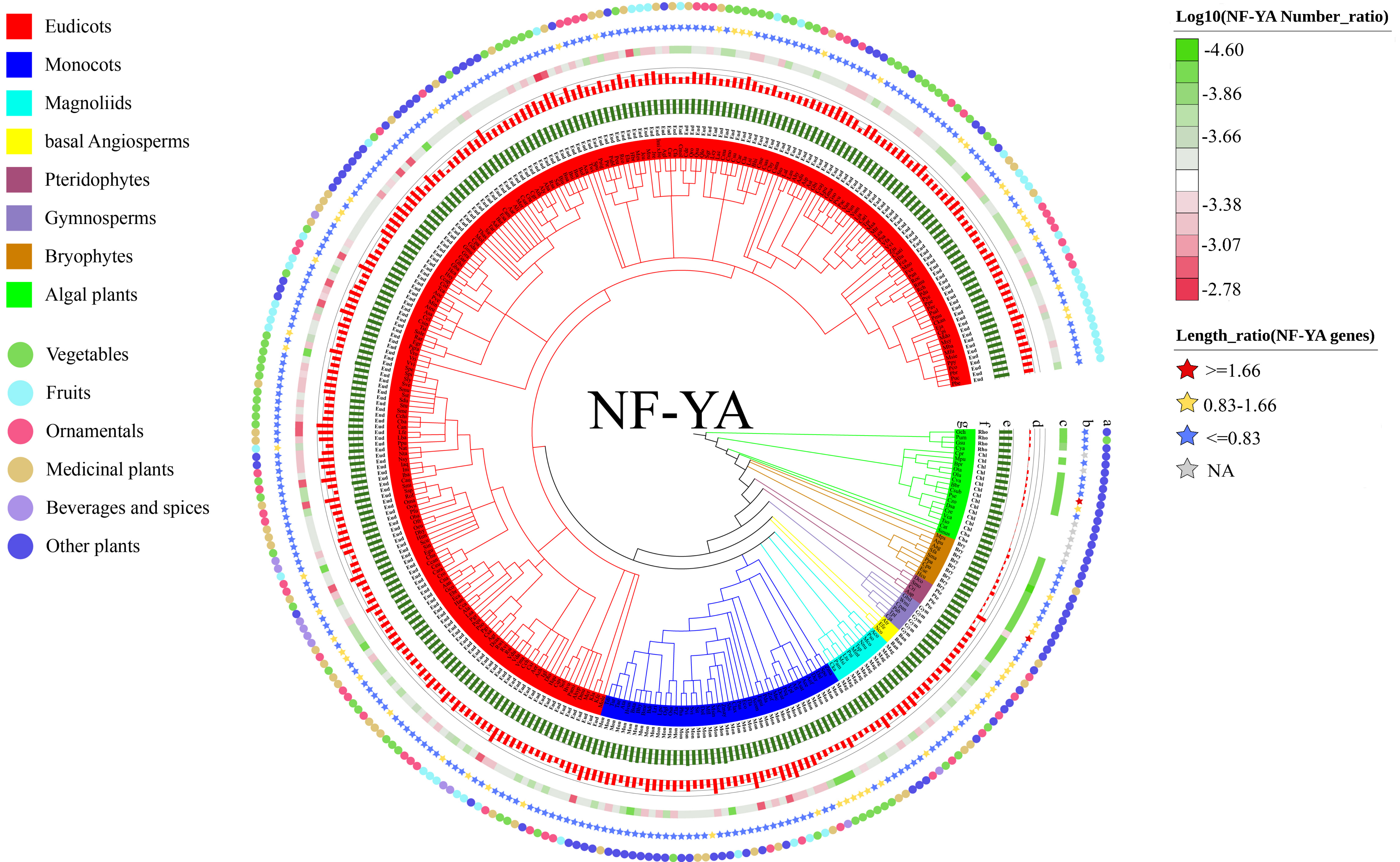
Eudicots
Monocots
Magnoliids
basal Angiosperms
Pteridophytes
Gymnosperms
Bryophytes
Algal plants
Vegetables
Fruits
Ornamentals
Medicinal plants
Beverages and spices
Other plants
NF-YA
Log10(NF-YA Number_ratio)
-4.60
-3.86
-3.66
-3.38
-3.07
-2.78
Length_ratio(NF-YA genes)
>=1.66
0.83-1.66
<=0.83
NA
a
b
c
d
e
f
g
