# Supplementary material for: Large-scale comparative analysis of the nuclear factor-Y transcription factors across 320 horticultural and other plants
Source: Hortic Res. 2025 Nov 4;13(2):uhaf304. doi: 10.1093/hr/uhaf304 (PMC12936444; doi:10.1093/hr/uhaf304)
Supplement: Web_Material_uhaf304 [file web_material_uhaf304.zip › Fig S5.pdf]

Motif	Logo	E-value	Width	Annotation
1	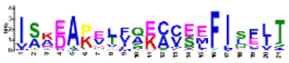	2.9e-1398	21	CBFA_NFYB_domain, Histone-fold
2	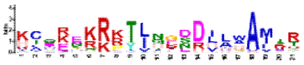	5.2E-1367	21	CBFA_NFYB_domain, Histone-fold
3	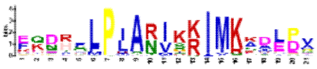	2.2E-1120	21	Histone-fold
4	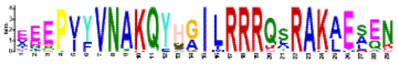	9.4E-671	29	NFYA, CCAAT-binding_factor_CS
5	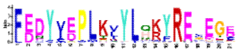	5.3E-560	21	-
6	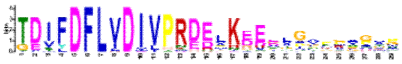	1.2E-487	29	-
7	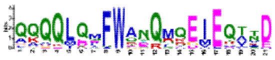	2.2E-271	21	-
8	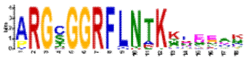	1.3E-267	18	NFYA
9	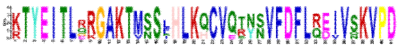	1.1E-159	41	Histone-fold
10	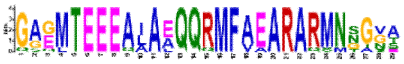	3.5E-105	29	Dr1-like
11	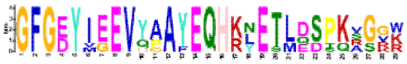	1.4E-091	29	-
12	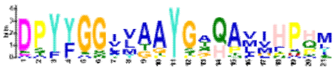	9.1E-099	21	-
13	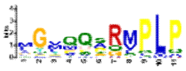	1.3E-043	11	-
14	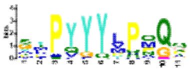	6.1E-044	11	-
15	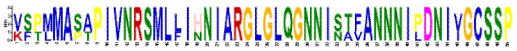	5.0E-039	50	-
16	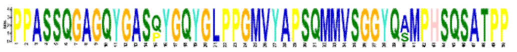	1.4E-038	50	-
17	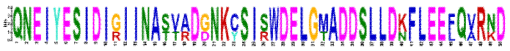	1.1E-042	50	-
18	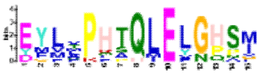	2.1E-032	15	-
19	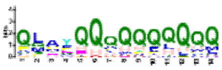	3.4E-045	14	-
20	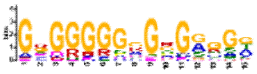	8.9E-031	15	-
