# Supplementary material for: Large-scale comparative analysis of the nuclear factor-Y transcription factors across 320 horticultural and other plants
Source: Hortic Res. 2025 Nov 4;13(2):uhaf304. doi: 10.1093/hr/uhaf304 (PMC12936444; doi:10.1093/hr/uhaf304)
Supplement: Web_Material_uhaf304 [file web_material_uhaf304.zip › Fig S8.pdf]

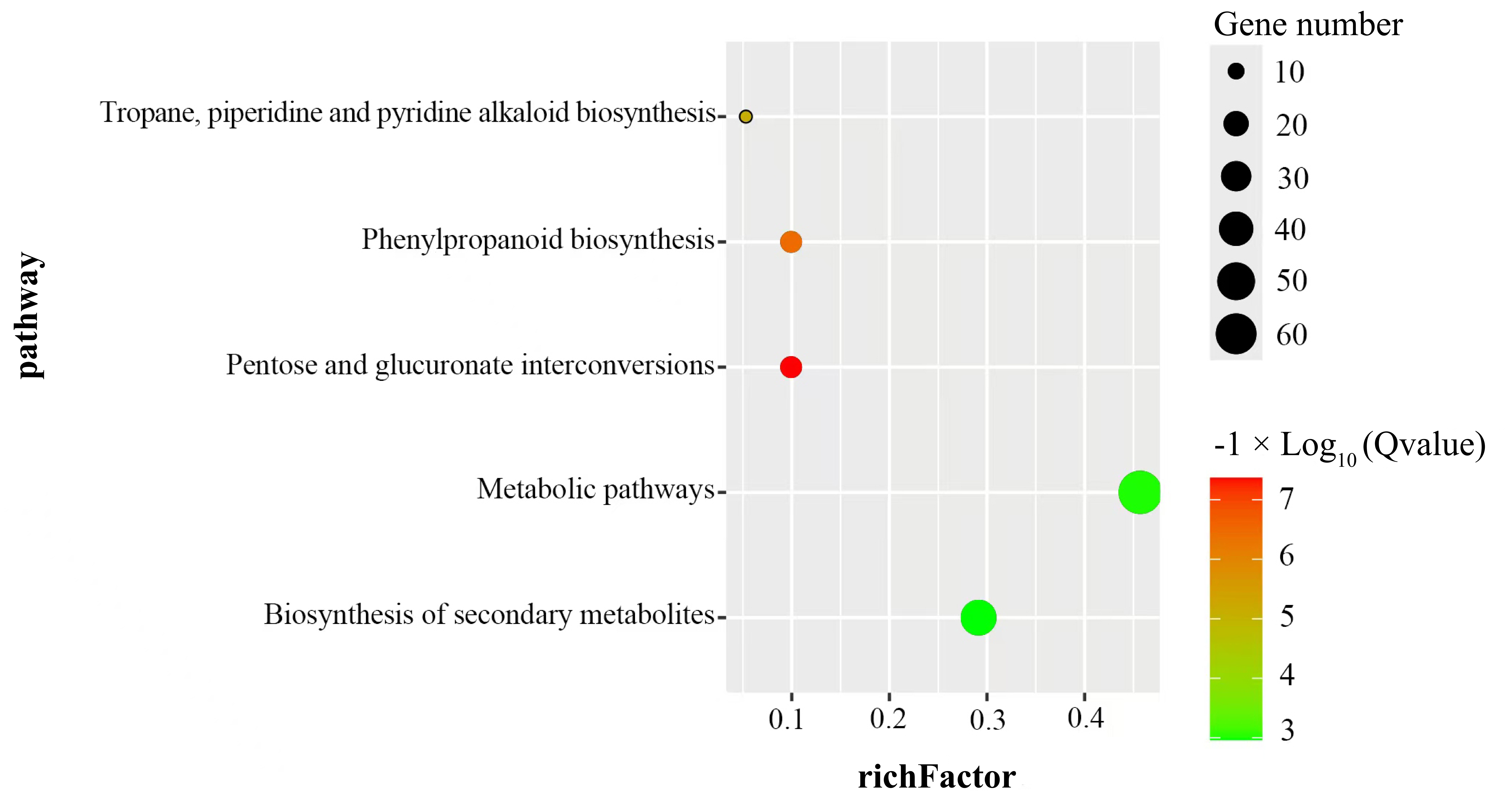

pathway
Tropane, piperidine and pyridine alkaloid biosynthesis
Phenylpropanoid biosynthesis
Pentose and glucuronate interconversions
Metabolic pathways
Biosynthesis of secondary metabolites
0.1
0.2
0.3
0.4
richFactor
Gene number
10
20
30
40
50
60
$-1 \times Log_{10}$ (Qvalue)
7
6
5
4
3
