# Supplementary figures and images for: Large-scale comparative analysis of the nuclear factor-Y transcription factors across 320 horticultural and other plants
Source: Hortic Res. 2025 Nov 4;13(2):uhaf304. doi: 10.1093/hr/uhaf304 (PMC12936444; doi:10.1093/hr/uhaf304)

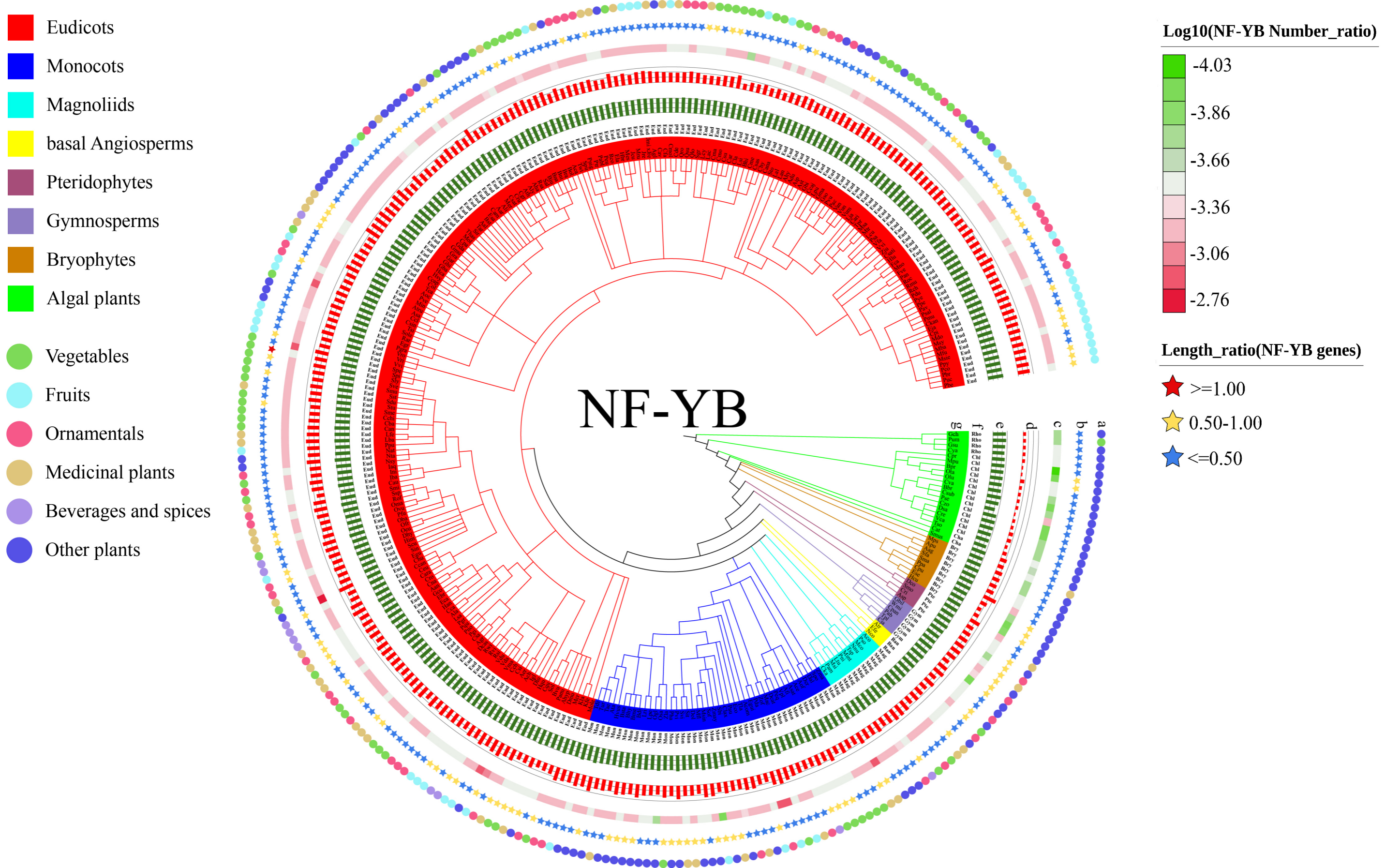

Supplement: Web_Material_uhaf304 [file web_material_uhaf304.zip › Fig S2.pdf]

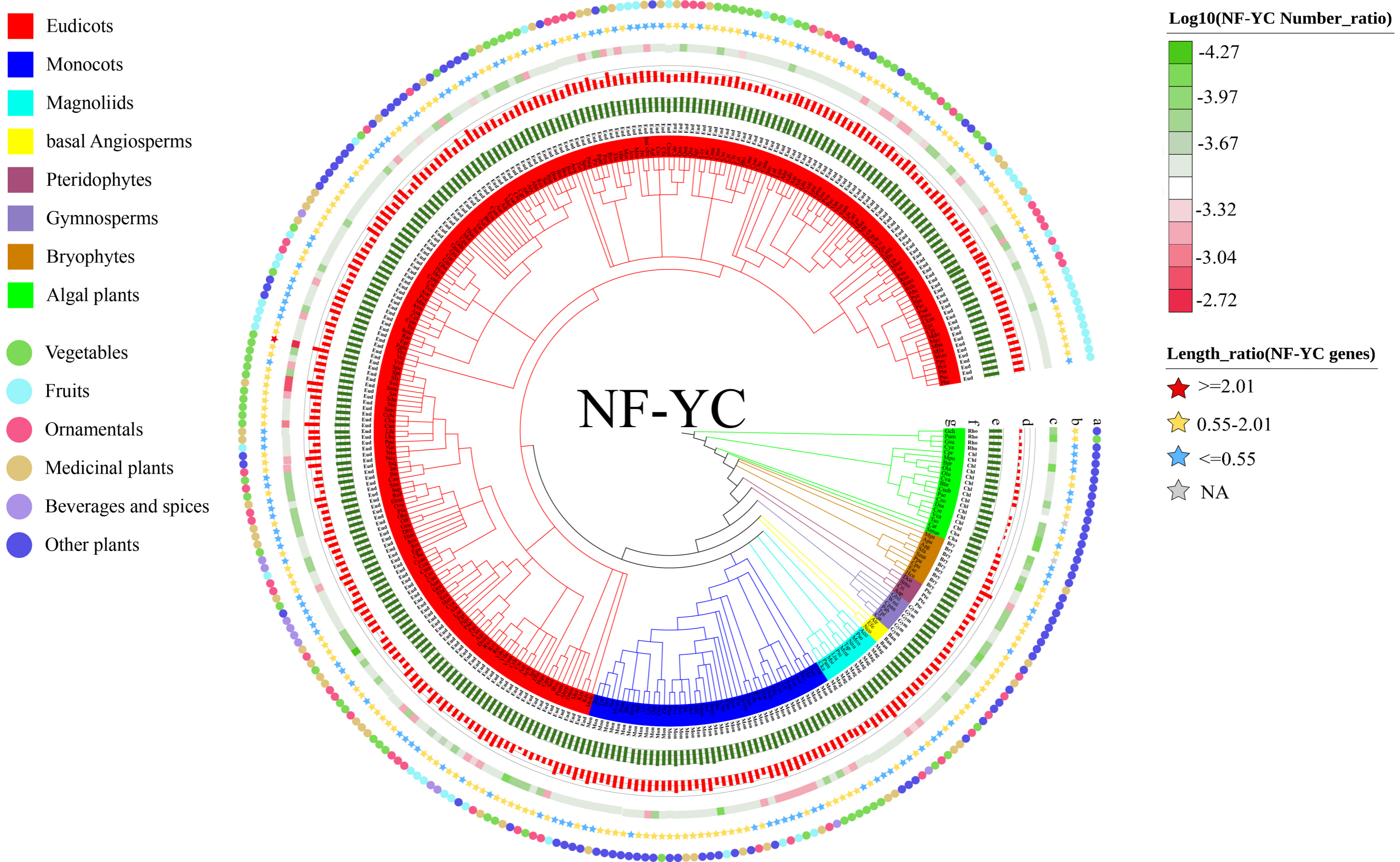

Supplement: Web_Material_uhaf304 [file web_material_uhaf304.zip › Fig S3.pdf]

The number of NF-Y family genes

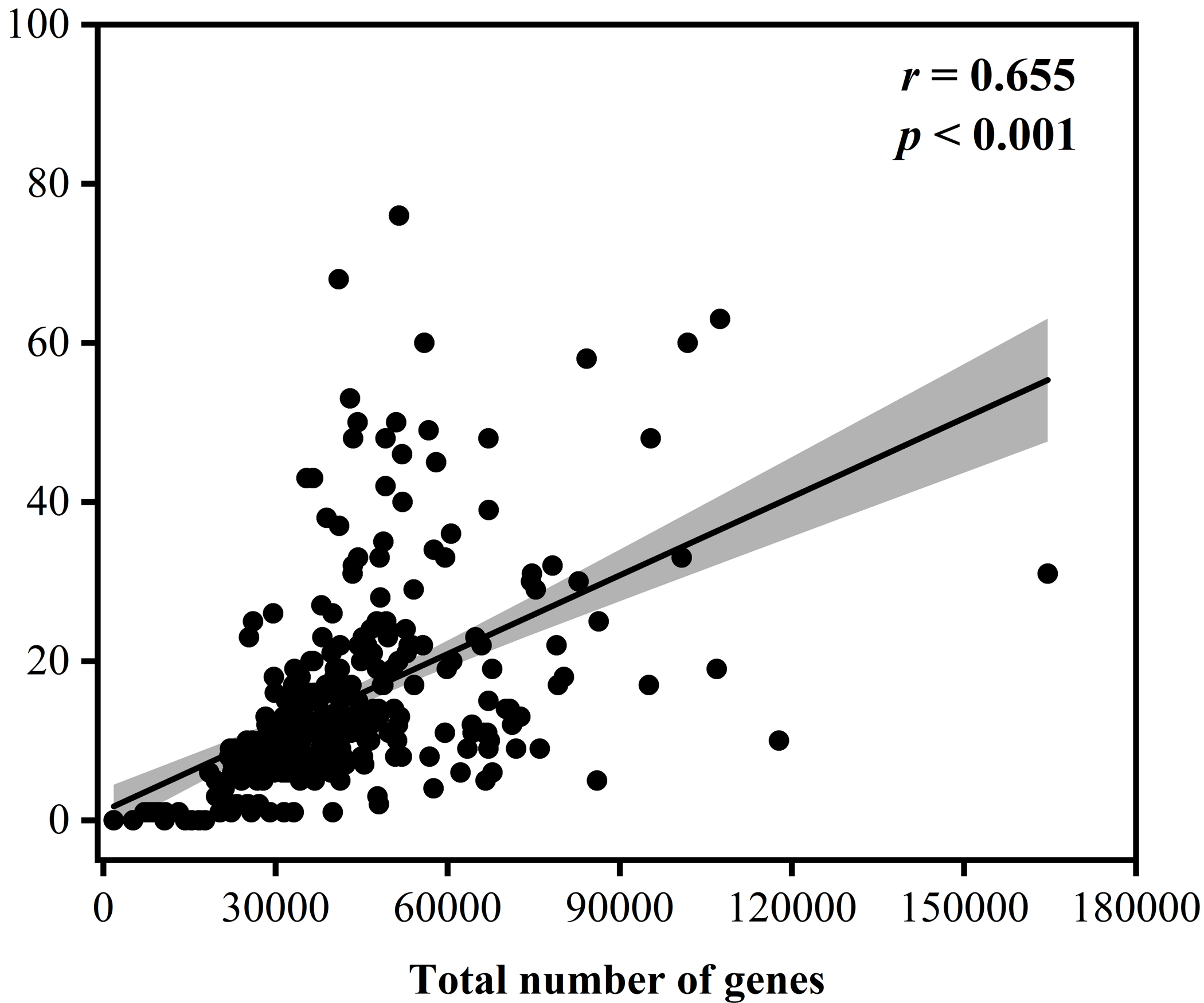

Supplement: Web_Material_uhaf304 [file web_material_uhaf304.zip › Fig S4.pdf]

NF-YA

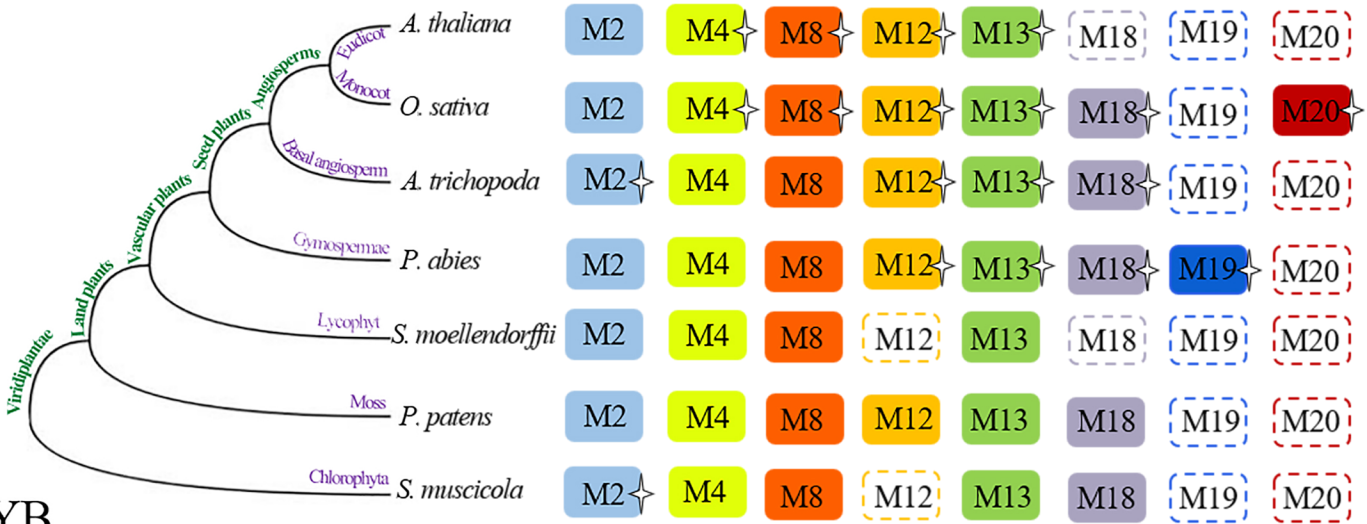

NF-YB

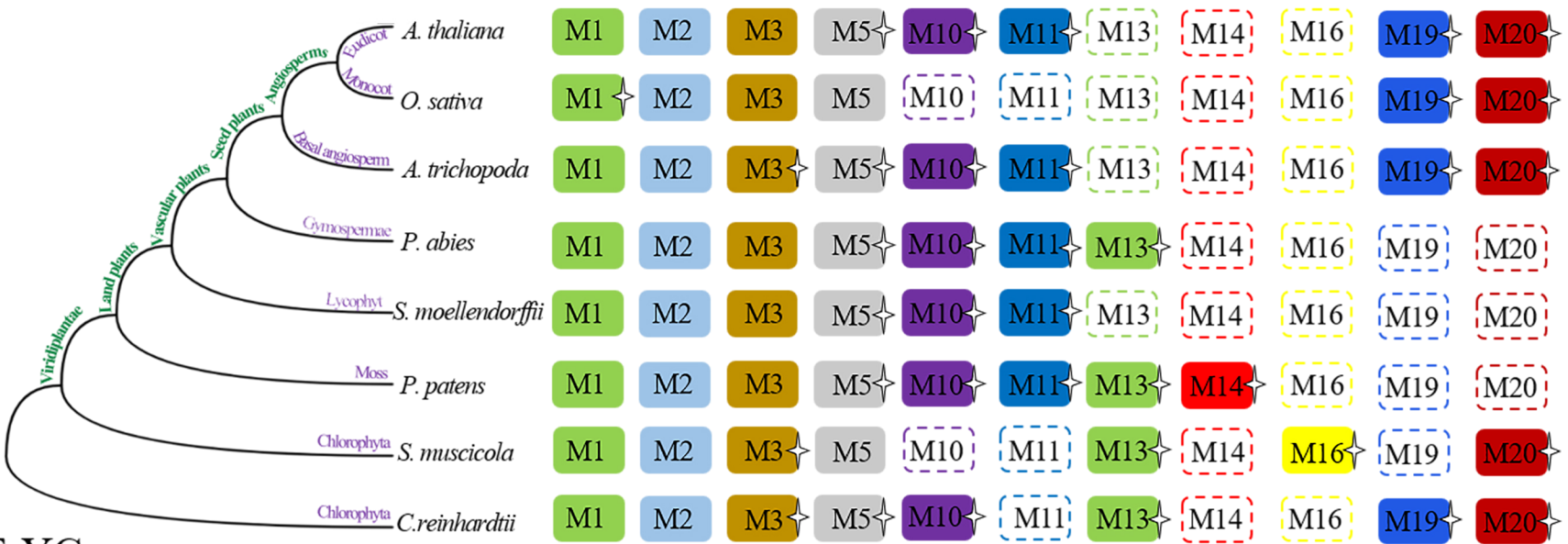

NF-YC

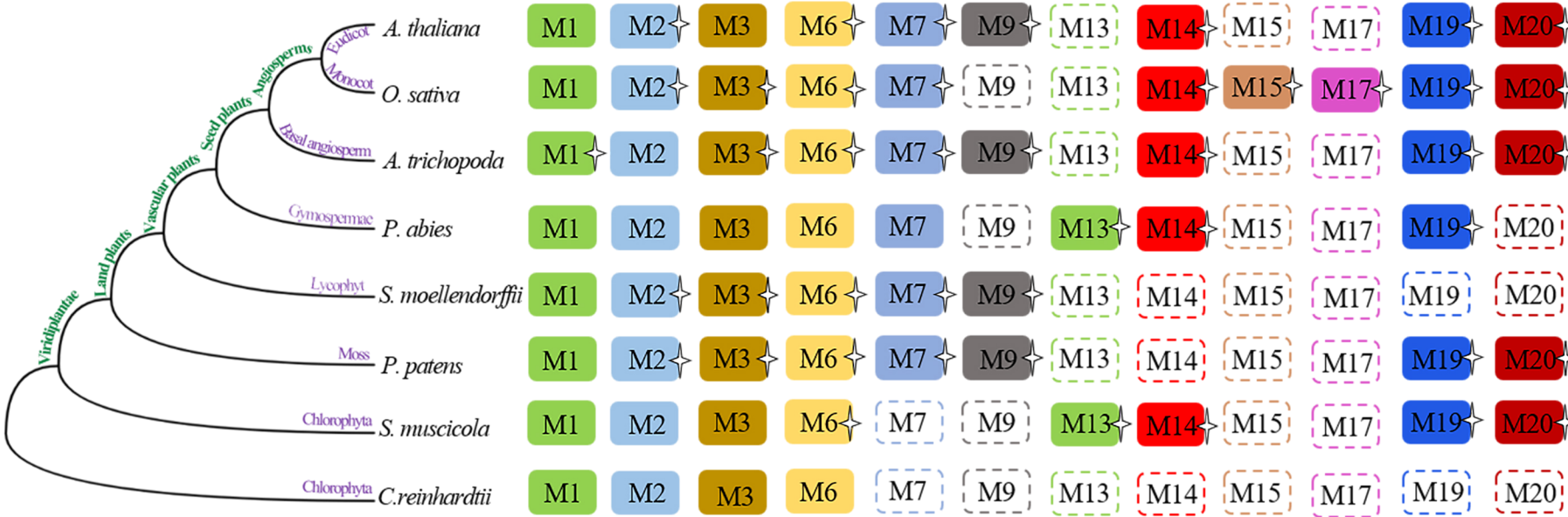

Supplement: Web_Material_uhaf304 [file web_material_uhaf304.zip › Fig S6.pdf]

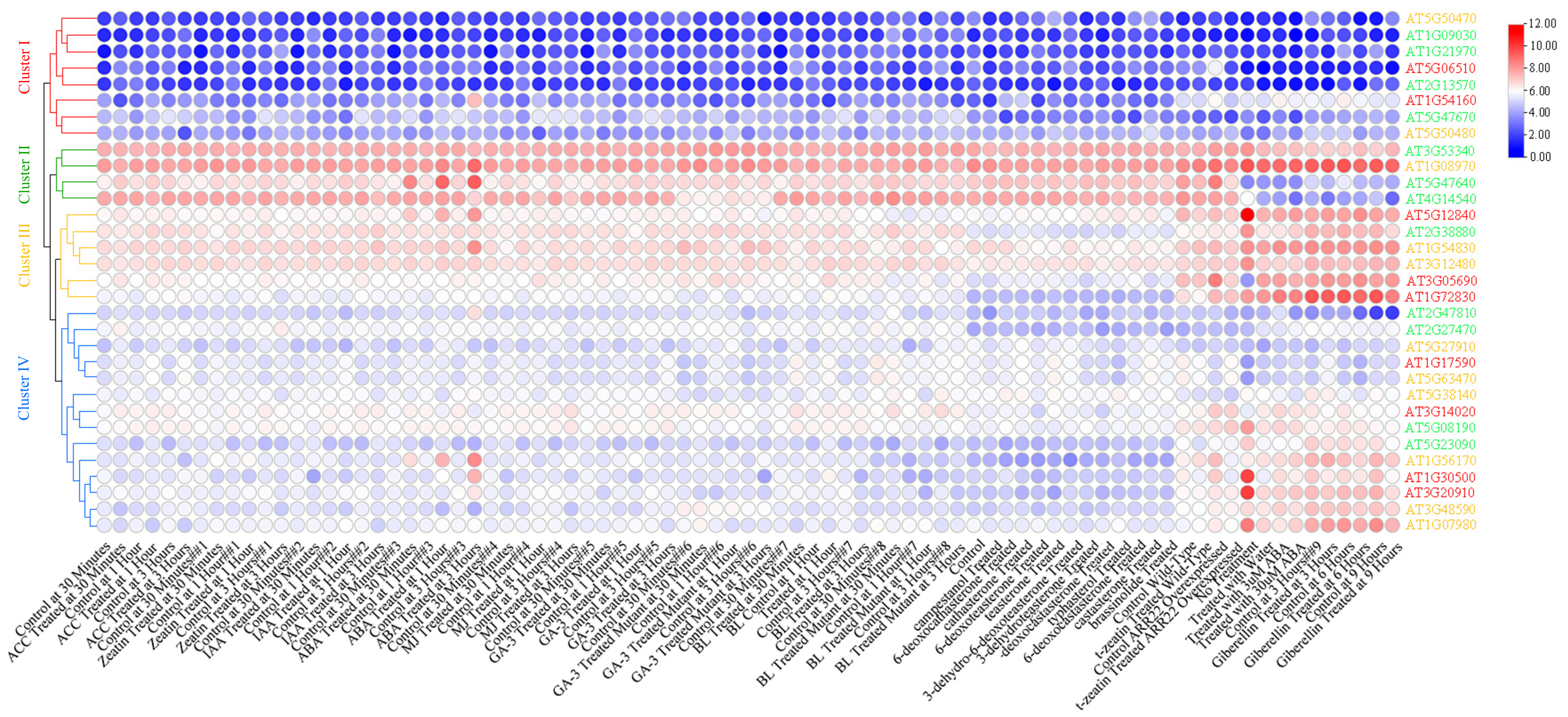

Supplement: Web_Material_uhaf304 [file web_material_uhaf304.zip › Fig S7.pdf]
